# Genome‐wide association study reveals candidate loci on ECA1 and ECA9 for withers height in Friesian horses

**DOI:** 10.1111/age.70049

**Published:** 2025-10-15

**Authors:** Marije J. Steensma, Harmen P. Doekes, Martijn F. L. Derks, Bart J. Ducro

**Affiliations:** ^1^ Animal Breeding and Genomics Wageningen University and Research Wageningen The Netherlands; ^2^ Koninklijke Vereniging ‘het Friesch Paarden‐Stamboek’ Drachten DP The Netherlands

**Keywords:** ECA1, growth, GWAS, horse, withers height

## Abstract

In Friesian horses, withers height is an important trait as a minimum has been set to be eligible to the studbook. Several loci for withers height have been identified in horses. However, withers height has not been studied in the Friesian horse. Therefore, our aim was to identify loci associated with withers height in the Friesian horse population. We performed a genome‐wide association study using 70 K SNP data of 2192 Friesian horses. We found ECA1 and ECA9 to be significantly associated with withers height, explaining 19.6% and 3.5% of the phenotypic variance, respectively. In other horse breeds, the *LCORL/NCPAG* locus on ECA3 showed the strongest association with withers height, but here we found that the best‐associated SNP for that locus is nearly fixed in Friesian horses for the allele associated with small size. Moreover, we observed a clear decline followed by a marked increase in average withers height of the Friesian horse over time, probably owing to shifts in its primary use over the course of the years. Additionally, the frequency of the best‐associated SNP on ECA1 has increased over time. Together, our study showed that ECA1 and ECA9 are associated with withers height in Friesian horses. Further studies should be performed to confirm candidate causal mutations.

In Friesian horses, withers height is an important trait as a minimum has been set to be eligible to the studbook. In other horse breeds, QTL on several chromosomes have been identified for withers height (Frischknecht et al., 2015; Gurgul et al., 2019; Makvandi‐Nejad et al., 2012; Reich et al., 2024; Signer‐Hasler et al., 2012). For the majority of horse breeds, the most significant associations with withers height have been reported for a QTL on ECA3 close to the *LCORL/NCAPG* genes that explained ~11–18% of the phenotypic variance (Gurgul et al., 2019; Makvandi‐Nejad et al., 2012; Metzger et al., 2013; Reich et al., 2024; Signer‐Hasler et al., 2012; Tetens et al., 2013). Still, withers height in Friesian horses has not yet been studied. Given the genetic distinctiveness of the Friesian horse compared with other horse breeds and its small effective population size (*N*_*e*_) (Schurink et al., 2019; Steensma et al., 2024), the genetic background for withers height might differ in Friesian horses. Therefore, our aim was to identify loci associated with withers height in Friesian horses.

In total, 2192 Friesian horses born between 1996 and 2021 (Figure S1) had both genotypes and phenotypes available. All genotype and phenotype data in this study were retrieved from ‘Het Koninklijk Friesch‐Paarden Stamboek’ (KFPS). Withers height was measured at inspection between 1999 and 2024, at a mean age of 1330 days (SD = 460 days), and ranged from 154 to 179 cm with a mean of 163.7 cm (SD = 3.3 cm). The horses were all genotyped in 2023 or 2024 as part of routine genotyping with the GGP Equine 70 K Plus BeadChip (Neogen/Illumina), including 71 618 SNPs based on EquCab3.0. Only autosomal SNPs were kept and duplicated SNPs, SNPs with MAF <0.01 and/or a call rate <0.9 were discarded, resulting in a final set of 41 835 markers. All horses had a genotype call rate >0.9, and therefore, all horses were included in the analysis.

We ran a mixed linear model based association analysis using the genome‐wide complex trait analysis (gcta) v1.93.3beta2 software (Yang et al., 2011). The model was:

$$\begin{array}{c} {\mathrm{WH}}_{\mathrm{ijk}}=\mu +{\mathrm{Age}}_{i}+{\mathrm{Sex}}_{j}+\beta_{1}\mathrm{PC}1+\ldots +\beta_{10}\mathrm{PC}10\\ \;+\beta_{11}\text{BYear}+\beta_{12}\mathrm{SNP}+g_{k}+e_{\mathrm{ijk}}, \end{array}$$

where WH_*ijk*_ = withers height of the animal *k*, *μ* = intercept, age_*i*_ = fixed effect of the *i*th age class at the time of inspection (*i* = 2.5–3 years old, 3–3.5 years old, 3.5–4 years old, 4–4.5 years old, 4.5–5 years old, 5–5.5 years old, and above 5.5 years old), sex_*j*_ = fixed effect of the *j*th sex class (*j* = male or female), PC1–PC10 = principal components reflecting the population structure, BYear = covariate of birth year, SNP = SNP genotype indicator variable coded as 0, 1, or 2 (representing the number of minor alleles), *β*_1_–*β*_12_ = respective regression coefficients, *g*_*k*_ = additive genetic effect that is assumed to follow a normal distribution of *N*(0,*Gσ*_a_^2^), where *G* is the genomic relationship matrix (GRM) and *e*_*ijk*_ = random residual effect. To adjust for multiple testing, a Bonferroni adjustment was applied to set the genome‐wide significance threshold. Manhattan plots were created using r v4.3.1 (Team RC, 2016).

Genome‐wide association study results showed two regions that were significantly associated with withers height. The first region was on ECA1 between 52 401 961 and 66 261 252 bp and the second region was on ECA9 between 76 863 026 and 77 493 377 bp (Figure 1). The best‐associated SNP on ECA1 was Affx‐102 480 508 located at 1:57621065 (*p* = 3.0 × 10^−41^) (Figure 1, Table S1, Figure S2). Horses with the genotypes A/A (*N* = 1102), A/G (*N* = 902) and G/G (*N* = 182) for this SNP had raw mean withers heights of 162.8 cm, 164.3 cm, and 166.2 cm, respectively (Figure 2a). For six horses, the genotype of the best‐associated SNP was unknown. The ±0.5 Mb region surrounding the best‐associated SNP on ECA1 was manually examined using jbrowse (Buels et al., 2016) and comprised several protein‐coding genes (Table S2). Within the significant region on ECA9, the only protein‐coding gene was the zinc finger and AT‐hook domain containing (*ZFAT*).

**FIGURE 1:**
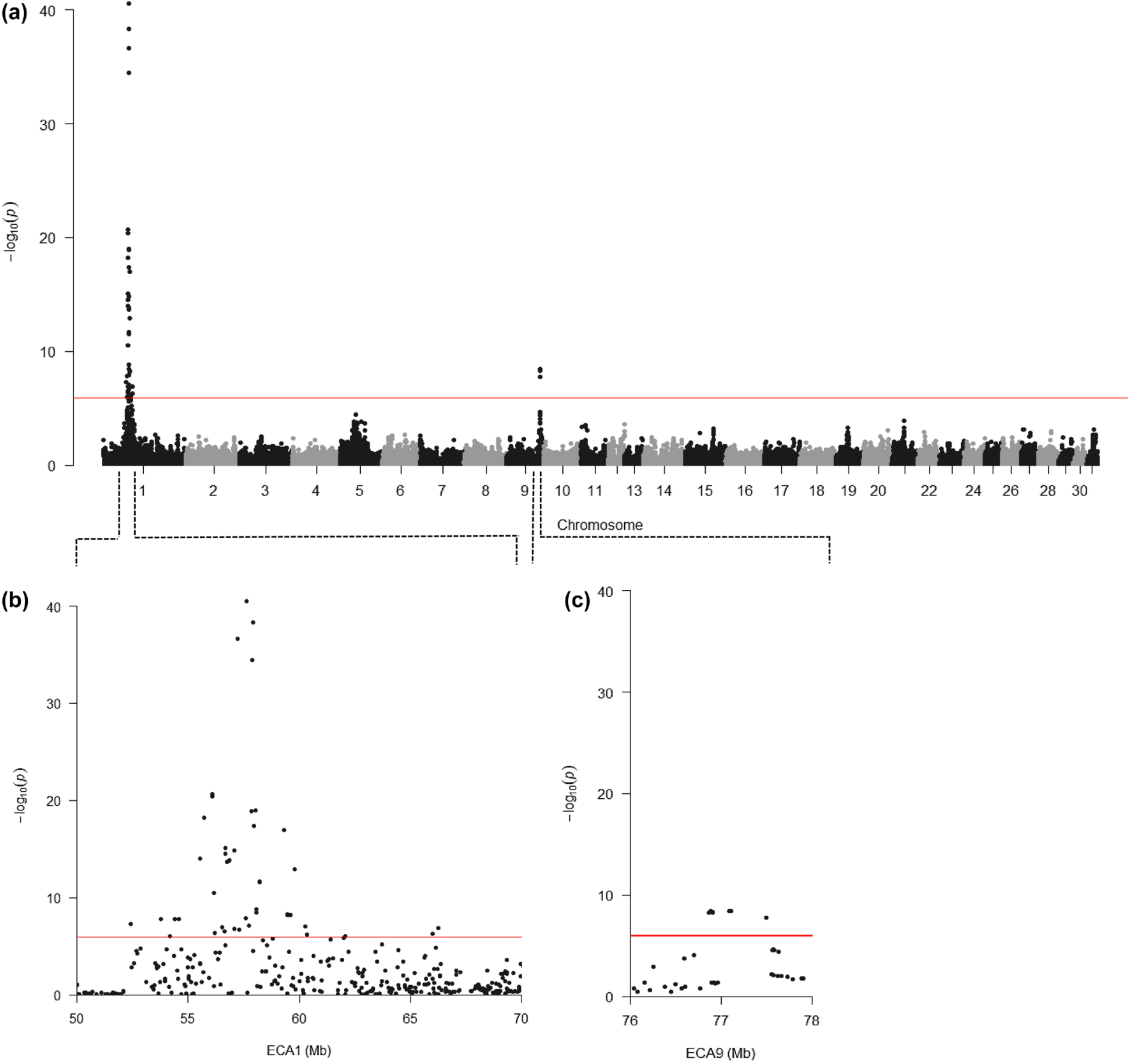
Manhattan plots of the −log_10_ *p*‐values from genome‐wide association study (mixed linear model based association analysis) for withers height in 2192 horses. (a) Manhattan plot of all chromosomes. (b) Manhattan plot of the significant region on ECA1. (c) Manhattan plot of the significant region on ECA9. A Bonferroni adjustment (*p* = 1.2 × 10^−6^) was applied to set the genome wide‐ significance threshold (red lines). One SNP was non‐estimable and was therefore removed from the plot.

**FIGURE 2:**
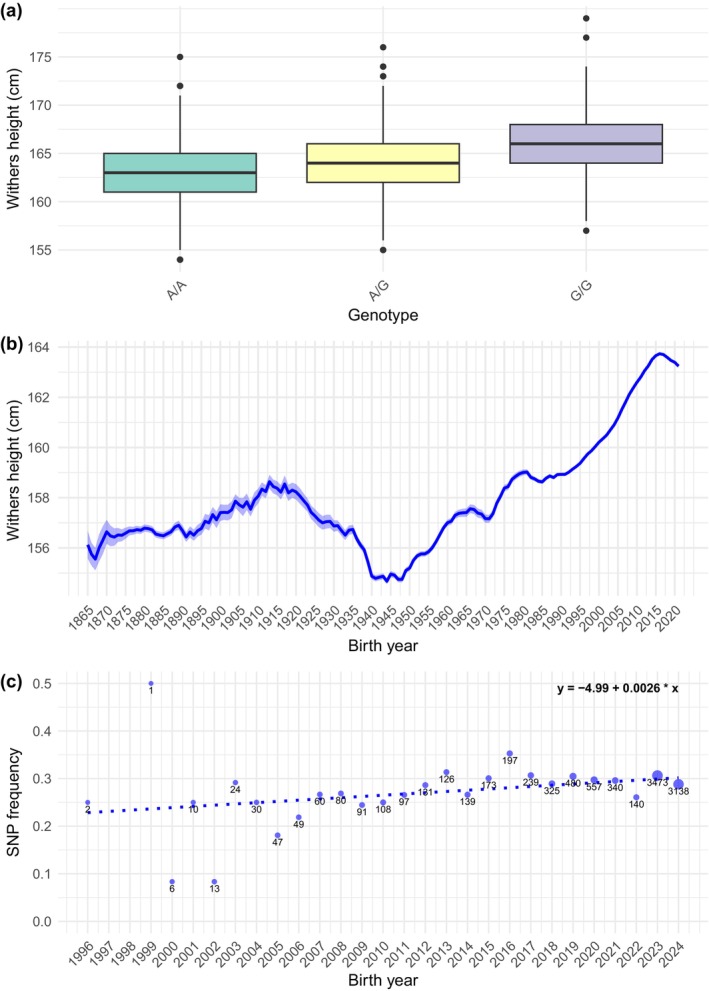
Overview of the best‐associated SNP Affx‐102 480 508 on ECA1. (a) Boxplot of withers height (cm) per genotype group of the best‐associated SNP Affx‐102 480 508. (b) Withers height (cm) of 50 089 Friesian mares born between 1863 and 2021, provided by the KFPS. The *y*‐axis represents withers height as moving average of 5 years (including 2 years before and after each birth year). Birth years on the *x*‐axis are grouped in overlapping 5 year intervals. For example, birth year 1865 on the *x*‐axis represents the withers height average (cm) for the period 1863–1867. The shaded area around the line represents ±1 standard error of the mean. (c) Frequency of the best‐associated SNP Affx‐102 480 508 per birth year for all available genotypes of 10 076 Friesian horses. The numbers at the dots represents the number of horses per birth year. The dotted line represents the weighted regression line based on the inverse of the variance 1/(SE^2^). The regression equation is shown in the upper right corner of the figure.

To assess the contribution of each chromosome to the total phenotypic variance, we built a separate GRM for each chromosome using GCTA v1.93.3beta2 (Yang et al., 2011). Then, a restricted maximum likelihood analysis was run in GCTA (Yang et al., 2011) with the option ‐‐mgrm. We found that ECA1 had the highest contribution to the phenotypic variance for withers height (19.6%) (Figure S4), with the best‐associated SNP on ECA1 accounting for 13.0% of the phenotypic variance, using the formula *Va*/*Vp*, where *Va* = 2*p*(1 − *p*)*b* ^2^ (Hivert et al., 2021) (*Va* = the additive genetic variance, *Vp* = the total phenotypic variance, *p* = the allele frequency and *b* = the allele substitution effect). The other chromosomes accounted for 0.001–4.6% of the phenotypic variance (Figure S4).

Furthermore, the whole genome sequence (WGS) data of 50 influential Friesian sires was available from the KFPS, allowing the detection of potential causal variants influencing withers height. Whole genome sequence data from paired‐end 150 bp reads were sequenced by BGI Genomics on DNBSEQ‐T7 (mean sequencing depth = 24.9×, range 12.8–50.0×) and aligned to EquCab3 (GCA_002863925.1) using bwa‐mem2 v2.2.1 (Vasimuddin et al., 2019). samblaster v.0.1.26 (Faust & Hall, 2014) was used to mark duplicates and samtools v1.17 (Li et al., 2009) to sort and index the BAM files. Variant calling was performed using the pipelines for SNP (population‐variant‐calling‐version2) and SV (population‐structural‐var‐calling‐smoove‐version2) calling with standard settings available at https://git.wur.nl/users/kim.lensing/projects. Variant consequences were predicted using bcftools/csq (Danecek & McCarthy, 2017).

To detect potential causal variants that might influence withers height, we looked for protein‐altering SNPs and structural variants (SVs) detected in the above‐mentioned WGS data that were in high linkage disequilibrium (LD) with the best‐associated SNP on ECA1 or ECA9. Therefore, similar to the study of Reich et al. (2024), LD was calculated between the best‐associated SNP for both ECA1 and ECA9 and SNPs/SVs detected in the above‐mentioned WGS data using plink v1.90b3.38 (Purcell et al., 2007) with the option ‐‐r^2^ and ‐‐ld‐window‐r^2^ 0.8 (Reich et al., 2024). This approach revealed seven moderate impact variants that were in high LD (*r* ^2^ > 0.8) with the best‐associated SNP on ECA1 (Table S3) and no moderate or high impact variants that were in high LD with the best‐associated SNP on ECA9. The seven variants on ECA1 were all missense variants within the genes *PBLD*, *RUFY2*, *TET1* and *STOX1*. One missense variant in *RUFY2* was predicted to be deleterious (SIFT = 0). Moreover, the missense variants in *PBLD* and *TET1* had a GERP score of 4.02 and 4.68, where variants with a GERP score above 4 are often assumed to be deleterious (Huber et al., 2020) (Table S3).

Interestingly, the missense variant in *TET1* (NM_030625.3:c.4091T>A) with a SIFT score of 0.12 has been previously described as being associated with withers height in German Warmblood horses (Reich et al., 2024). Additionally, *TET1* has been associated with height in humans (Benonisdottir et al., 2016; Buniello et al., 2019) and mutant mice displayed a smaller size at birth (Dawlaty et al., 2011). Moreover, *RUFY2* has been previously linked to height in humans (Sakaue et al., 2021). The other two genes have not been previously associated with height. *PBLD* could be a candidate gene as it has a positive effect on negative regulation of the transforming growth factor beta receptor signaling pathway (Alliance of Genome Resources, 2024). Previous studies found *MYPN* on ECA1 to be associated with withers height in horses (Brooks et al., 2018; Reich et al., 2024). Those studies found two missense variants in *MYPN*, which were also found in the Friesian horse; however, they were in low LD with the best‐associated SNP on ECA1 (*r* ^2^ = 0.27). Together, the identified missense variants in our study may constitute causal mutations influencing withers height in horses. Still, further analyses are needed to validate the candidate causal mutations.

We furthermore analyzed the withers height of 50 089 Friesian mares born between 1863 and 2021 (Figure 2b). We included only mares in this analysis, as the sex‐ratio has varied substantially over the years (Figure S3). After a slight increase from the 1860s until 1913, the average withers height decreased from 158.6 cm in 1915 to 154.7 cm in 1947, followed by a steady increase to 163.7 cm in 2017, and a slight drop to 163.2 cm in 2021 (Figure 2b). These fluctuations probably reflect changes in the primary use of the Friesian horse. Namely, around 1913, the Friesian horse was close to extinction, prompting a shift in breeding focus from an elegant carriage horse to a more compact and heavier‐built draft horse suitable for agricultural work. Following the onset of mechanization in the 1950s, the demand for draft horses declined, and breeding efforts shifted towards a more versatile riding horse (KFPS, 2025), contributing to the observed increase in withers height. Moreover, we used genotype data from 10 076 (GGP Equine 70 K Plus BeadChip (Neogen/Illumina)) Friesians to examine whether the allele frequency of the best‐associated SNP changed over the years by performing a weighted linear regression (1/SE^2^). Despite most animals having been born in recent years, we observed that the frequency of the allele associated with an increased withers height significantly increased by 0.0026 per year (*p* < 0.01) (Figure 2c).

The candidate region on ECA1 has been described in a few studies. The ECA1 region was found to be significantly associated with withers height in German Warmblood horses (Reich et al., 2024), American Belgian Draft horses (Brooks et al., 2018) and French jumping horses (Ricard et al., 2023). Moreover, ECA1 was found to explain a large part of the phenotypic variance for withers height in Franches–Montagnes horses (11%) (Signer‐Hasler et al., 2012) and German Warmblood horses (6%) (Tetens et al., 2013), but no significant peaks were found.

The significant region we found on ECA9 has been previously described in several horse breeds (Gurgul et al., 2019; Makvandi‐Nejad et al., 2012; Signer‐Hasler et al., 2012). This region comprises the *ZFAT* gene, however, no moderate or high impact variants in this gene were found to be in high LD with the best‐associated SNP on ECA9, which suggests the causal variant might be regulatory. Still, *ZFAT* is a good candidate gene to affect withers height as it was found to be associated with height in humans (Lango Allen et al., 2010; N'Diaye et al., 2011; Takeuchi et al., 2009).

Here, we saw that the ‘short’ T allele located on ECA3:107374136 bp is nearly fixed (MAF = 0.0005) in the Friesian horse population. In contrast, the American Belgian Draft horse is fixed for the ‘tall’ C allele (Brooks et al., 2018). This SNP near the *LCROL/NCAPG* genes was the best‐associated SNP for withers height in other horse breeds (Gurgul et al., 2019; Makvandi‐Nejad et al., 2012; Metzger et al., 2013; Reich et al., 2024; Signer‐Hasler et al., 2012; Tetens et al., 2013). However, in Friesian horses, the frequency of this SNP is too low to determine any possible association with a phenotypic effect.

To conclude, ECA1 and ECA9 were significantly associated with withers height in Friesian horses, explaining 19.6% and 3.5% of the phenotypic variance, respectively. In other horse breeds, a locus on ECA3 near *LCORL/NCAPG* has been previously associated with withers height, but we found that the Friesian horse is nearly fixed for the allele associated with small size in horses. Friesian horses first showed a decrease followed by an increase in average withers height over the years, which could have led to an increase in allele frequency of the best‐associated SNP on ECA1. Further research is needed to confirm causal mutations on ECA1 and ECA9.

## AUTHOR CONTRIBUTIONS

**Marije J. Steensma:** Conceptualization; methodology; software; data curation; investigation; funding acquisition; writing – original draft; writing – review and editing; project administration; resources; formal analysis; validation; visualization. **Harmen P. Doekes:** Conceptualization; writing – review and editing; supervision. **Martijn F. L. Derks:** Conceptualization; writing – review and editing; supervision. **Bart J. Ducro:** Conceptualization; writing – review and editing; supervision; project administration; funding acquisition.

## CONFLICT OF INTEREST STATEMENT

The authors declare that they have no competing interests.

## FUNDING INFORMATION

This study was conducted as part of the TKI PPS project ‘BehoudenPaard’ (No. 4164023400).

## ETHICS STATEMENT

The biological material used in this study was collected as part of routine data collection from the KFPS, and not specifically for the purpose of this project. Therefore, approval of an ethics committee was not mandatory. Blood sample collection was done by a licensed vet following the ‘Code of Good Veterinary Practice’. No animals were euthanized/sacrificed or anesthetized for this study. Sample collection was conducted strictly in line with Dutch law on the protection of animals (Gezondheids‐ en welzijnswet voor dieren).

## Supporting information


Figure S1.

Table S1.

## Data Availability

Seventy thousand genotypes of the significant regions on ECA1 and ECA9 (±3 Mb) and WGS variants (SNP and SV) in and surrounding the significant regions on ECA1 and ECA9 (±3 Mb) are available at the Open Science Framework repository: https://osf.io/xw5p7. Full genotype and sequence data are available from the Koninklijk Friesch Paarden Stamboek, but restrictions apply to the availability of these data, which were used under license for the current study, and so are not publicly available.
