# Supplementary material for: Genome‐wide association study reveals candidate loci on ECA1 and ECA9 for withers height in Friesian horses
Source: Anim Genet. 2025 Oct 15;56(5):e70049. doi: 10.1111/age.70049 (PMC12522178; doi:10.1111/age.70049)
Supplement: Supplementary file 1 — Figure S1. [file AGE-56-0-s002.docx]

**Supporting Figs.**

**
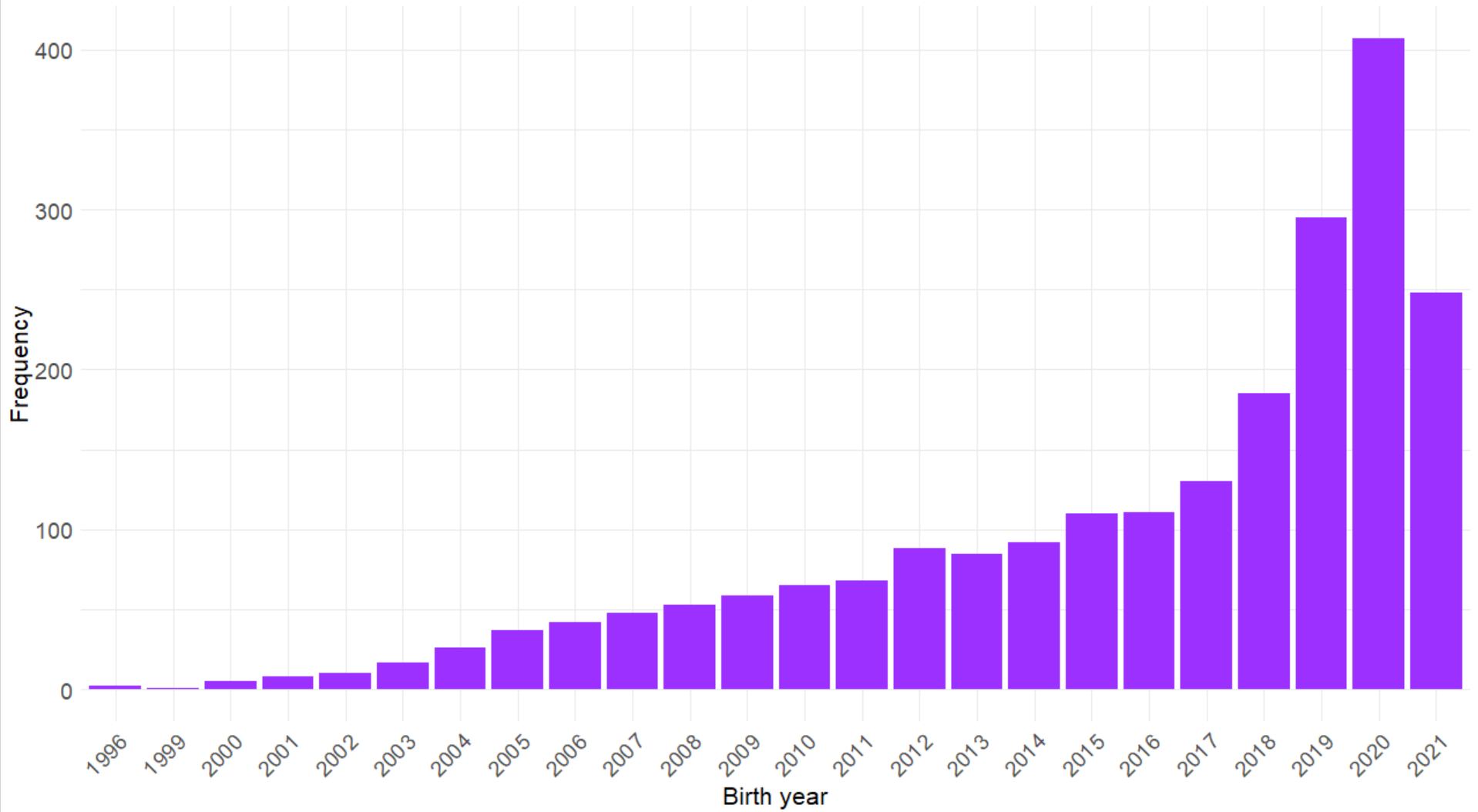
**

**Fig. S1. The number of genotyped horses (N = 2,192) shown per birth year.**


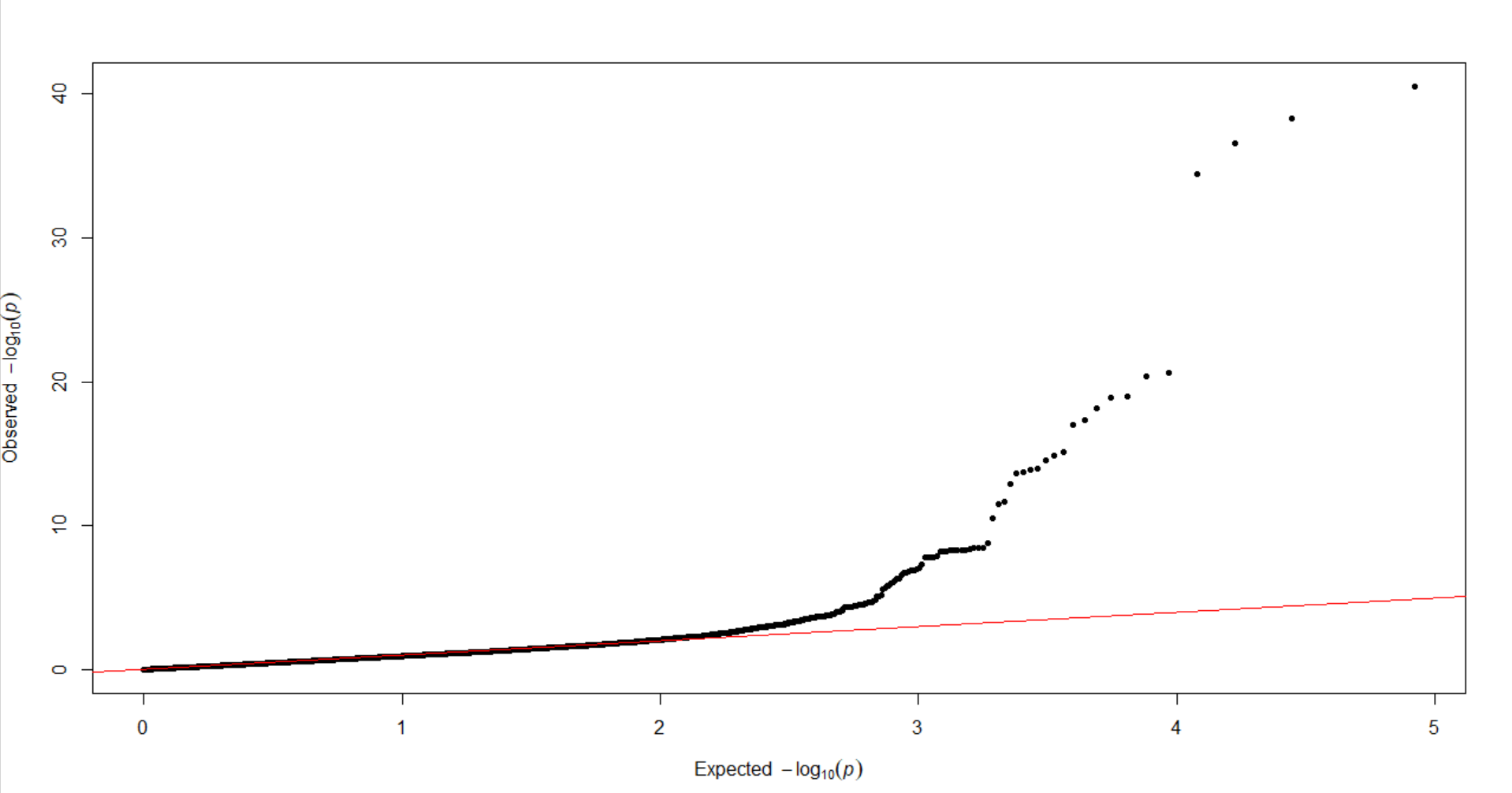


**Fig. S2.** **Q-Q plot of the observed versus expected -log_10_ P-values from a genome-wide association study (MLMA) for withers height in 2,192 Friesian horses.** The expected distribution (solid red line) and the observed −log_10_ P-values plotted against the expected −log_10_ P-values (black dots) are shown.


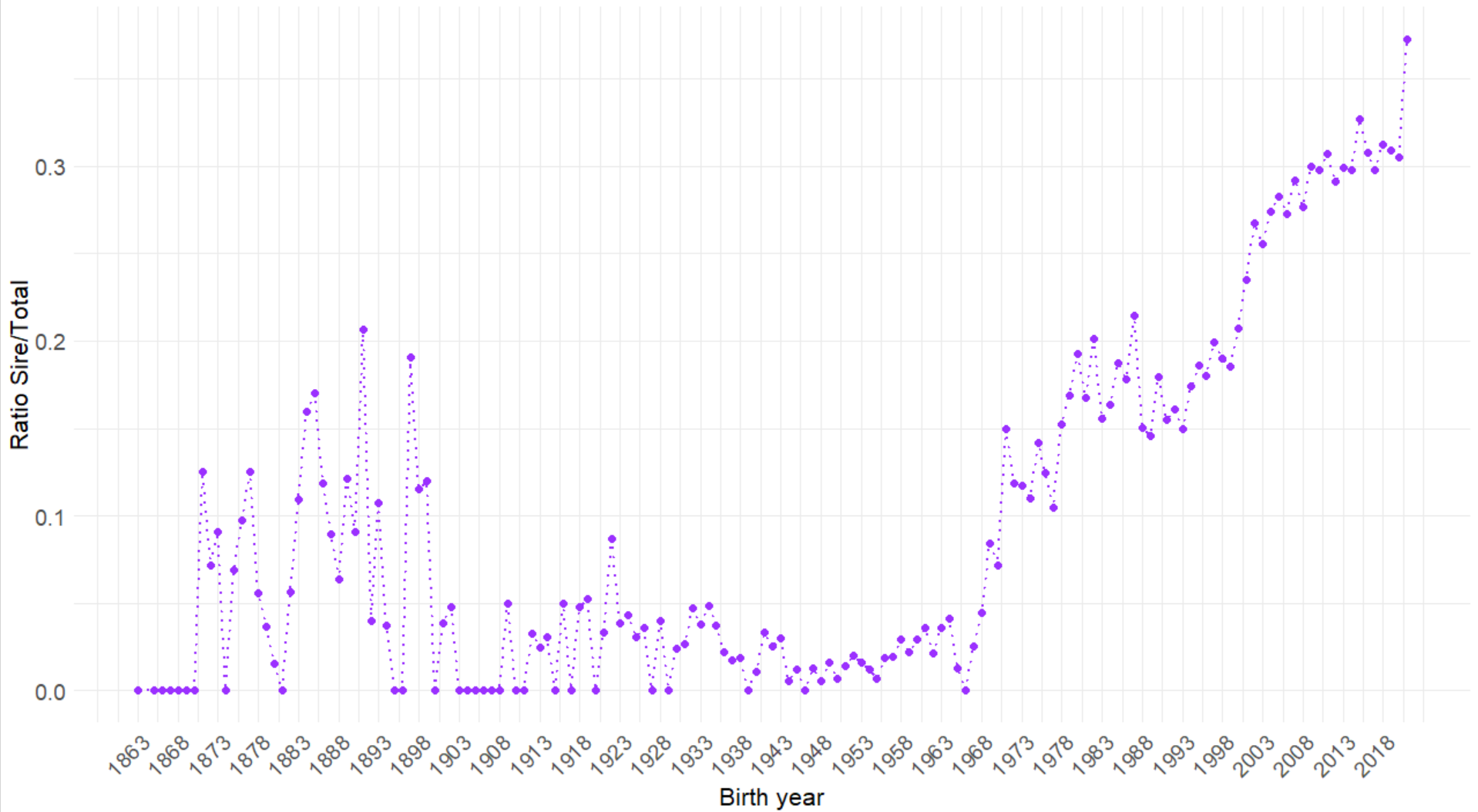


**Fig. S3.** Sex ratio sire/total of all the horses which have a phenotype on withers height (N = 64,517).


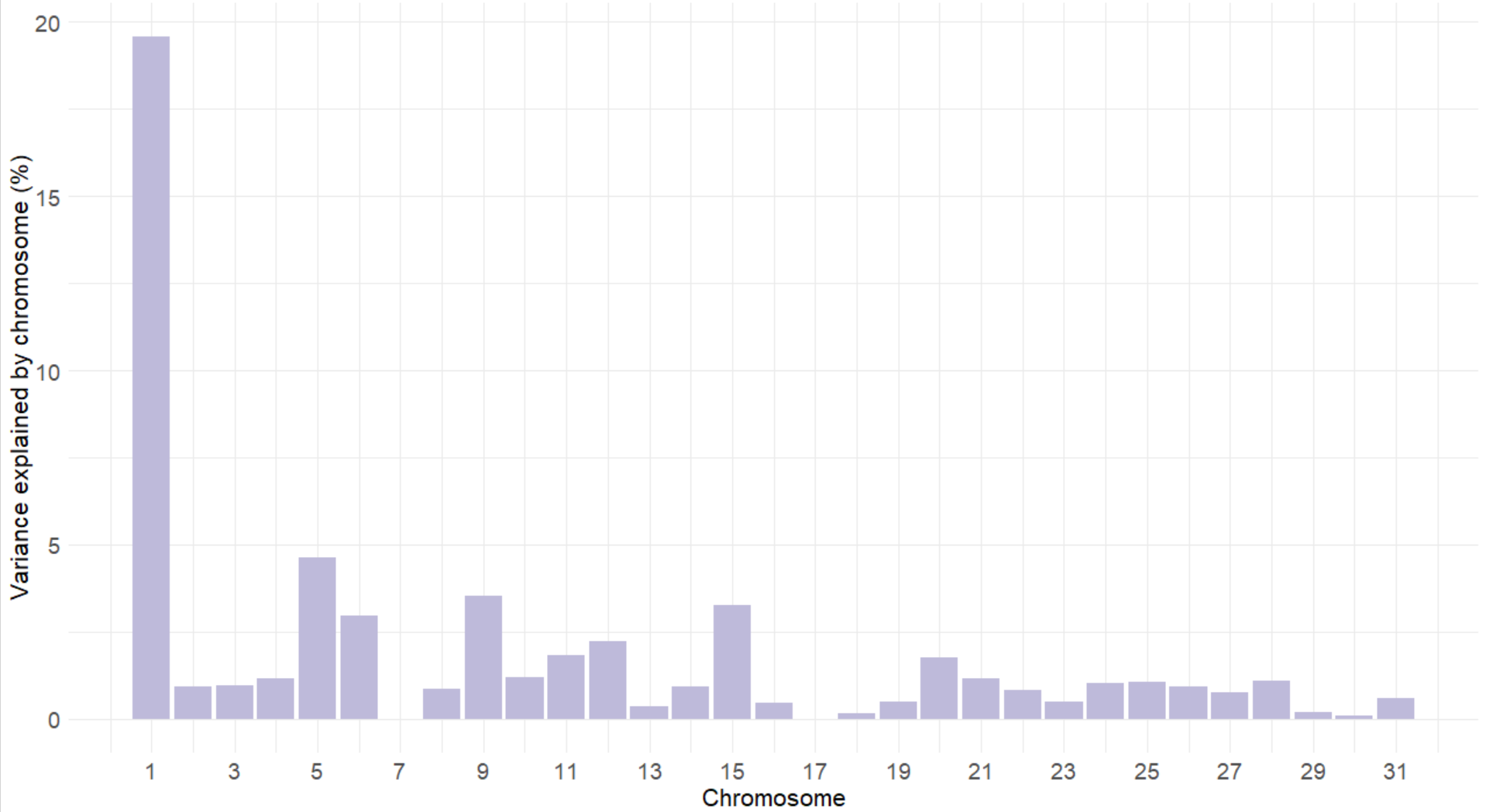


**Fig. S4.** Estimated phenotypic variance (%) explained by each of the 31 autosomes. In total, the SNP-based heritability for withers height was 55.4%, assuming a mean covariance of 0 between the estimated chromosome effects.
